# Royal Naval Medical Service

**Published:** 1914-09-05

**Authors:** 


### THE PUBLIC SERVICES.

### ROYAL NAVAL MEDICAL SERVICE.

*Qualification.*—1. Every candidate for admission into the Medical Department of the Royal Navy must be not under 21 nor over 28 years of age on the day of the commencement of the competitive examination. He must produce an extract from the register of the date of his birth, or, in default, a declaration made before a magistrate stating the date of birth.

2. He must declare: (1) His age and date and place of birth; (2) that he is of pure European descent, and the son of either natural born British subjects or of parents naturalised in the United Kingdom; (3) that he labours under no mental or constitutional disease or weakness, nor any other imperfection or disability which may interfere with the most efficient discharge of the duties of a medical officer in any climate; (4) that he is ready to engage for general service at home or abroad if required; (5) whether he holds, or has held, any commission or appointment in the Public Services; (6) that he is registered under the Medical Act, giving the date of his registration as a medical student or of his beginning professional study; (7) whether he has been previously examined for entry into the Naval Service, and, if so, when.

3. The certificates of registration and birth must accompany the declaration, which is to be filled up and returned as soon as possible, addressed to the Director-General, Medical Department, Admiralty, S.W., before the candidate can be allowed to compete for an existing vacancy.

4. The candidate will be examined as to his physical fitness by a Board of Naval Medical Officers.

5. Candidates who have served in the Officers' Training Corps will be credited with additional marks at the entrance examination in the following proportion: Those in possession of Certificate A, 1 per cent.; those in possession of Certificates A and B, 2 per cent. of the maximum number of marks allotted.

*Examination.*—1. Entrance examination held twice yearly in London in the following subjects: (*a*) Medicine, including medical pathology and therapeutics; (*b*) surgery, including surgical pathology and clinical surgery. The examination will be partly written and partly practical, marks being allotted under the following scheme:—

| | |
|---|---|
| **Medicine.** | |
| Paper ... ... ... ... ... ... | 400 |
| Clinical ... ... ... ... ... ... | 400 |
| Oral ... ... ... ... ... ... | 400 |
| | 1,200 |
| **Surgery.** | |
| Paper ... ... ... ... ... ... | 400 |
| Clinical ... ... ... ... ... ... | 400 |
| Oral ... ... ... ... ... ... | 400 |
| | 1,200 |

No candidate will be considered eligible unless he obtains 50 per cent. of the marks in each subject.

Successful candidates will be given appointments as acting surgeons in the Royal Navy, and will then enter upon a six months' course of training.

The first two months will be passed at the Naval Medical School, Greenwich. Here they will receive instruction in tropical medicine, pathology, and hygiene.

The remaining four months will be passed at Haslar, and will be devoted to the study of naval hygiene, recruiting, physical training, diving and submarine work, radiography, anæsthetics, dentistry, etc.

At the conclusion of the Greenwich and Haslar courses examinations will be held, and the marks gained in these, added to those gained at the entrance examination, will determine the seniority of the commissions granted to the successful candidates as Surgeons, R.N.

2. A further examination is held for the rank of staff-surgeon at Greenwich and London twice yearly, and follows on the course of instruction described below.

*Post Graduate Courses.*—1. Prior to presenting themselves for the examination for qualification for the rank of staff-surgeon officers are provided with a complete course of instruction of five months' duration at the Royal Naval Medical School, Greenwich, the "Dreadnought" Hospital, Greenwich, and other civil hospitals in London, as may be arranged from time to time.

This course of instruction comprises: (*a*) Clinical medicine and clinical surgery; (*b*) operative surgery; (*c*) anæsthetics (practical); (*d*) ophthalmology; (*e*) clinical pathology; (*f*) hygiene; (*g*) throat, nose, and ear diseases; (*h*) skiagraphy.

2. A course of three months' duration for officers of not less than fourteen years' seniority is arranged for twice yearly, and is directed to affording them opportunities of acquiring special knowledge.

3. In addition, courses of instruction of about six weeks' duration in clinical work and hospital administration are arranged for at each of the home ports for the benefit of those officers who may be in the ports at the time.

There are no formal examinations at the end of these courses, but they have a certain bearing on the progress of officers, as reports concerning them are transmitted to headquarters.

*Distinctions.*—(*a*) Prizes given at end of acting surgeons' course of instruction, viz.: A gold and silver medal and three surgical pocket-cases; (*b*) Gilbert Blane medal (this gold medal is awarded annually to the medical officer who obtains the highest aggregate marks at the half-yearly examinations for promotion to staff-surgeon); (*c*) Chadwick Naval Prize (£100, once in five years, for distinguished work in naval hygiene); (*d*) King's honorary physicians and surgeons.

*Promotions.*—Promotion to staff-surgeons and fleet-surgeons takes place at the end of eight and sixteen years respectively—provided the qualifying examination for staff-surgeon has been passed. In addition, special promotion will be made at their Lordships' discretion as a reward for distinguished service or conspicuous professional merit.

Accelerated promotion will be granted to officers who at the qualifying examination for staff-surgeon obtain at least 75 per cent. of the total marks.

Promotion to the ranks of deputy surgeon-general and surgeon-general are made by selection from the ranks of fleet-surgeons and deputy surgeons-general respectively.

*Withdrawals.*—Officers will be allowed to withdraw, at the discretion of their Lordships, with gratuities on the following scale: After four years' service, £500; after eight years' service, £1,000; after twelve years' service, £1,500; and after sixteen years' service, £2,250.

For retired pay and widows' pensions see the Regulations for the Naval Medical Service.

Officers joining the Service at the present time can feel assured of practically continuous employment.

